# Shared network pattern of lung squamous carcinoma and adenocarcinoma illuminates therapeutic targets for non-small cell lung cancer

**DOI:** 10.3389/fsurg.2022.958479

**Published:** 2022-10-03

**Authors:** Piaopiao Li, Xuemei Kuang, Tingting Zhang, Lei Ma

**Affiliations:** ^1^ College of Life Science, Shihezi University, Shihezi, Xinjiang Uyghur Region, China; ^2^ The First Affiliated Hospital, College of Medicine, Shihezi University, Shihezi, China

**Keywords:** non-small cell lung cancer, lung squamous carcinoma, lung adenocarcinoma, co-expression network, prognostic markers

## Abstract

**Background:**

Non-small cell lung cancer (NSCLC) is a malignant tumor with high mortality. Lung squamous carcinoma (LUSC) and lung adenocarcinoma (LUAD) are the common subtypes of NSCLC. However, how LUSC and LUAD are compatible remains to be elucidated.

**Methods:**

We used a network approach to find highly interconnected genes shared with LUSC and LUAD, and we then built modules to assess the degree of preservation between them. To quantify this result, *Z*-scores were used to summarize the interrelationships between LUSC and LUAD. Furthermore, we correlated network hub genes with patient survival time to identify risk factors.

**Results:**

Our findings provided a look at the regulatory pattern for LUSC and LUAD. For LUSC, several genes, such as *AKR1C1*, *AKR1C2*, and *AKR1C3*, play key roles in regulating network modules of cell growth pathways. In addition, *CCL19*, *CCR7*, *CCL21*, and *LY9* are enriched in LUAD network modules of T lymphocyte-related pathways. LUSC and LUAD have similar expressed gene expression patterns. Their networks share 46 hub genes with connectivity greater than 0.9. These genes are correlated with patient survival time. Among them, the expression level of *COL5A2* in LUSC and LUAD is higher than that in normal tissues, which is closely related to the poor prognosis of LUSC and LUAD patients.

**Conclusion:**

LUSC and LUAD share a network pattern. *COL5A2* may be a risk factor in poor prognosis in LUSC and LUAD. The common landscape of LUSC and LUAD will help better define the regulation of NSCLC candidate genes and achieve the goals of precision medicine.

## Introduction

Lung cancer is one of the fastest-growing malignancies in the world in terms of morbidity and mortality (1). Non-small cell lung carcinoma (NSCLC) accounts for more than 85% of lung cancer patients (2). Advanced NSCLC has a poorer prognosis compared to small cell lung cancer (3). It is imperative to find early biomarkers to judge prognosis and guide treatment for NSCLC (2). Lung squamous carcinoma (LUSC) and lung adenocarcinoma (LUAD) are the most common subtypes of NSCLC (4). They differ in genetics, pathogenesis, biological behavior, treatment, and prognosis (5–8). Generally, LUAD grows more slowly and has a smaller mass than LUSC at the same stage, but LUAD tends to start to metastasize at an early stage (9, 10). LUSCs metastasize later and are usually diagnosed at an advanced stage (11). LUAD is insensitive to radiation and chemotherapy (12). The prognosis of patients is unsatisfactory, and the 5-year survival rate is less than 10% (13). However, both subtypes lack effective early diagnosis methods. Therefore, elucidating the molecular mechanisms of these two subtypes and finding new prognostic markers is of great significance for the prognosis of patients with NSCLC (14).

Comparative studies of cancer types based on common features (13) and individual distinct attributes can provide new insights into different cancers at the molecular level (15). Network analysis is an effective means to provide key insights into the relationship between gene expression levels and the different progression of cancers (16). To access how LUSC and LUAD are compatible, we built a network to find highly interconnected genes associated with them. In sum, LUSC and LUAD share a common gene expression pattern with 46 common hub genes in both networks. In addition, the *COL5A2* gene may be a major factor in poor prognosis in LUSC and LUAD. The common landscape of LUSC and LUAD may provide potential target genes for the diagnosis of NSCLC and provide a new insight into the precision therapy of LUSC and LUAD.

## Material and methods

### Data preparation

We downloaded 484 LUSC and 510 LUAD cohorts from the cBioPortal for Cancer Genomics (http://www.cbioportal.org/) (17). We then used R to standardize the dataset and determine the comparability of the data. By calculating the median absolute deviation, we selected the top 20% of genes shared by the two cancers. Then, according to the degree of sample aggregation estimated by the WGCNA package, 136 LUSCs and 184 LUADs were retained (Supplementary Table S1) (18). The two datasets were comparable when the data correlation between LUSCs and LUSCs was 0.46 (*P* < 0.01) (Supplementary Figure S1).

### Network analysis of weighted gene co-expression

We set genes as nodes and relationships between genes as edges to build a co-expression network using the WGCNA package (18). The construction steps of the network mainly included correlation matrix calculation, soft threshold selection, adjacency matrix calculation, heterogeneous matrix calculation, dynamic branch cutting, and module merging (19). We then identified the network modules.

### Module consensus between subtypes

We identified the consensus module between LUSC and LUAD. Module overlap degree referred to the common gene number between modules (20). We then generated *Z*-scores (20) using the permutation test in the WGCNA package to assess the preservation of LUAD genes in the LUSC module. The ranges 5 < *Z* < 10 and *Z* > 10 were considered moderate and highly preserved, respectively.

### Module eigengene and GO enrichment

Module eigengene (ME), the first principal component of modules, represents the feature expression mode of modules (21). Eigengene connectivity (KME) represents the Pearson correlation between genes (including genes not originally assigned to modules) and modules in the network. We calculated KME and *P* values for all genes in LUSC and LUAD, then ranked the KME values from largest to smallest and selected the top 100 genes for each module (*P* < 0.05). We performed a Gene Ontology (GO) enrichment analysis to select the top five enrichment terms for each module *via* the Clusterprofiler package (22).

### Hub gene screen

Based on *Z*-scores, we selected candidate hub genes with KME-module correlations greater than 0.9 in high-conservation modules. We then performed Spearman's rank correlation analysis between the expression of candidate hub genes and the overall survival time of patients. Finally, the online software Kaplan–Meier Plotter (23) was used to analyze the effect of gene expression on patient survival. Cancer samples were divided into two groups based on high and low expression levels of genes. Genes with significantly different survival curves between the two groups were thought to be closely related to the survival of cancer patients (19). We used Cox univariate analysis in the SURVIVAL and SURVMISER software packages to verify whether hub gene expression is a major prognostic factor.

### Validation of COL5A2

Based on the GEO database (https://www.ncbi.nlm.nih.gov/gds/), we obtained the LUSC and LUAD gene expression dataset (GSE134381), including 20 LUSC cancer samples and 20 normal samples, and 17 LUAD cancer samples and 17 normal samples. We evaluated the expression differences of hub genes in cancer and normal samples. We searched for chemical substances and human diseases that have regulatory relationships with hub genes in the Comparative Toxicogenomics Database (CTD) (24) and counted the number of corresponding files.

## Results

### Similarity of LUSC and LUAD network

**Figure 1:**
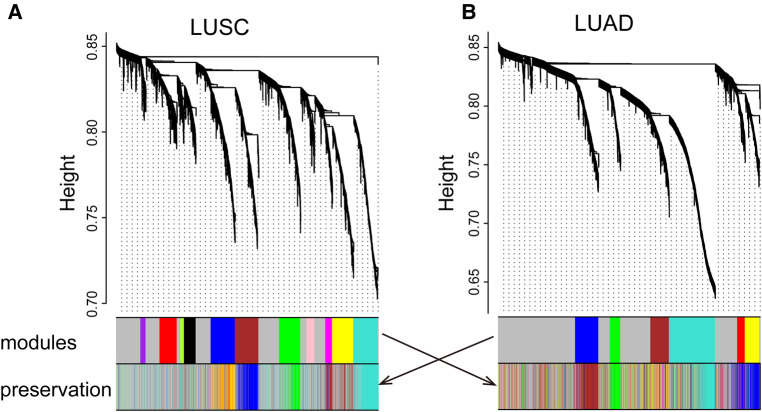
LUSC (**A**) and LUAD (**B**) networks. The upper panel shows a gene dendrogram obtained by mean linkage hierarchical clustering. The first color row underneath the dendrogram shows the module assignment determined by the dynamic tree cut. The second row shows the imposition of a subtype module onto another network. These module color labels in the second row are still grouped together corresponding to the first row, indicating good preservation. Note that for some modules, even with significant preservation, they cannot be seen in obvious grouping in the second dataset.

We identified 11 LUSC and 6 LUAD gene co-expression modules, respectively (Figure 1 and Supplementary Figures S1, S2). For visualization, we named modules with colors. Modules are clusters of densely interconnected genes that may be involved in a similar function. For example, the LUSC module “greenyellow” contains some genes, such as *AKR1C1*, *AKR1C2*, and *AKR1C3*, enriched in regulation of the extent of cell growth pathways. In addition, the LUAD module “yellow” consists of genes, such as *CCL19*, *CCR7*, *CCL21*, and *LY9*, that are enriched in T-lymphocyte-related pathways involved in T-cell activation, regulation of lymphocyte activation, and regulation of T-cell activation.

**Figure 2:**
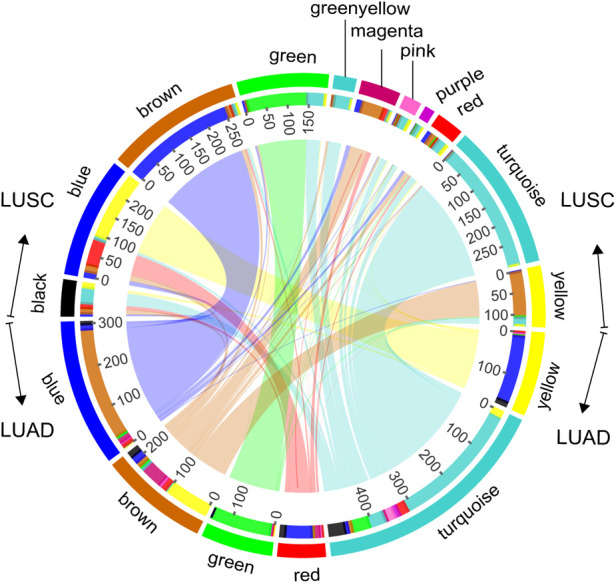
Module overlaps. Circles represent, from outermost to innermost, the color labels of modules of LUSC and LUAD, the number of genes, and internetwork overlapping.

Modules highlighted similar expression patterns of genes in LUSC and LUAD, respectively. We then assessed how well the modules in one cancer subtype are preserved in another cancer subtype. As a qualitative evaluation, we imposed modules from LUSC onto the network for dataset LUAD and vice versa (Figures 1A,B). Some modules from one cancer subtype are still assembled in another cancer, well preserved (Figure 2; Supplementary Table S2). There is a high degree of gene overlap within the module between LUSC and LUAD. For example, the LUSC module “turquoise” is contained in the LUAD module “turquoise,” and the LUSC module “brown” is corresponding to the LUAD module “blue.” To quantify this result, we used the *Z*-score to summarize how well the modules hold in each other (Table 1). In general, the higher the value of the *Z*-score, the more preserved the module is between cancer subtypes. The *Z*-scores of the six modules are greater than 10, indicating that the LUSC network is significantly similar to LUAD (Table 1).

**Table 1:** LUSC and LUAD network module.

LUSC module	Gene number	LUAD module	Gene number	Preservation *Z*-score
Turquoise^a^	354	Turquoise	670	44.715663
Blue	352	Yellow	208	21.557415
Brown	344	Blue	339	28.486611
Yellow	309	Brown	270	23.538526
Green	309	Green	152	10.620995
Red	251	Red	111	10.467881
Black	170	—	—	3.714300
Pink	115	—	—	3.543998
Magenta	99	—	—	2.992362
Purple	76	—	—	2.552492
Greenyellow	53	—	—	1.174076

^a^ Modules are named by color for visualization, corresponding to the color in Figures 1 and 2.

### Module function similarity

**Figure 3:**
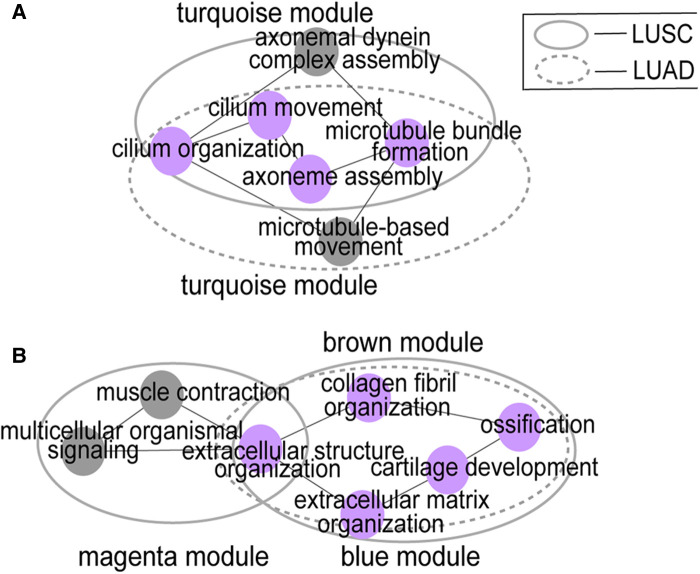
Module function similarity. (**A**) shows the functional overlap between LUSC module “turquoise” and LUAD module “turquoise”; (**B**) shows the functional overlap between LUSC module “brown” and LUAD module “blue”, the LUSC module “magenta” and the LUAD module “blue”. Big circles represent modules and small colored circles represent GO terms. The purple circles represent the inter-network overlap.

We explored the top five significant rich GO terms for each module. The functional overlap degree of modules between LUSC and LUAD corresponds to the degree of preservation of the module among them (Figure 3 and Supplementary Table S3). The higher the degree of preservation between the LUSC module and the LUAD module, the higher the functional overlap between them. For example, the LUSC module “turquoise” and the LUAD module “turquoise” have the highest *Z*-score, sharing 80% (4/5) of GO terms (Figure 3A). Furthermore, LUSC module “brown” and LUAD module “blue” are almost completely overlapping (Figure 2), which share common biological processes, such as extracellular matrix organization, extracellular structure organization, collagen fibril organization, ossification, and cartilage development (Figure 3B). In addition, low-preservation modules also have shared GO terms. For example, both the LUSC module “magenta” and the LUAD module “blue” are involved in extracellular structure in organization processes (Figure 3B).

### Hub genes

**Figure 4:**
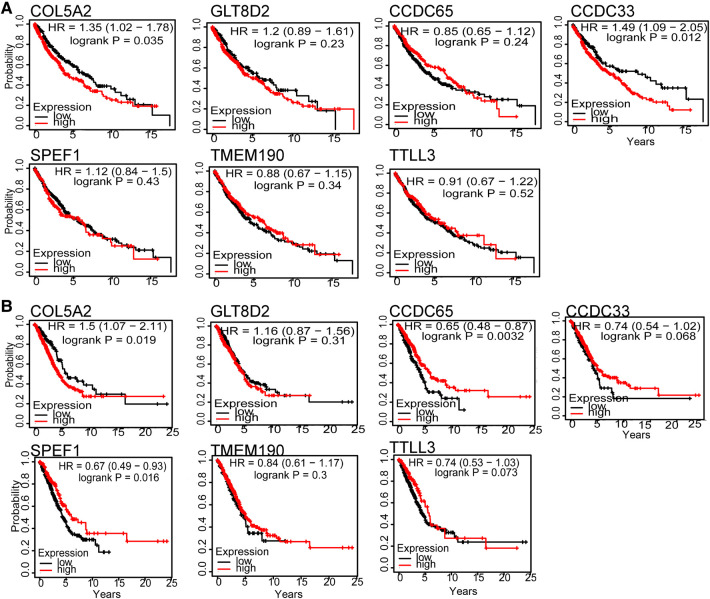
Hub genes survival curve for seven genes in LUSC (**A**) and LUAD (**B**). The horizontal axis is survival time, and the vertical axis is the overall survival rate.

The LUSC and LUAD networks share 46 hub genes with connectivity greater than 0.9. We then correlated these genes with patient survival time. The hub genes of *COL5A2*, *TTLL3*, *SPEF1*, *TMEM190*, *CCDC65*, *CCDC33*, and *GLT8D2* have the highest Spearman rank correlations. Furthermore, *COL5A2* is closely related to both LUSC and LUAD patients’ survival time (Figure 4), implying it may be a risk factor. The *COL5A2* gene may lead to both LUSC and LUAD patients' poor prognosis [LUSC *HR* = 1.35 (1.02–1.78), logrank *P* = 0.035; LUAD *HR* = 1.5 (1.07–2.11), logrank *P* = 0.019]. The higher the *COL5A2* expression, the worse the patient survival rate (Figure 4).

### Prognostic factor *COL5A2*

**Figure 5:**
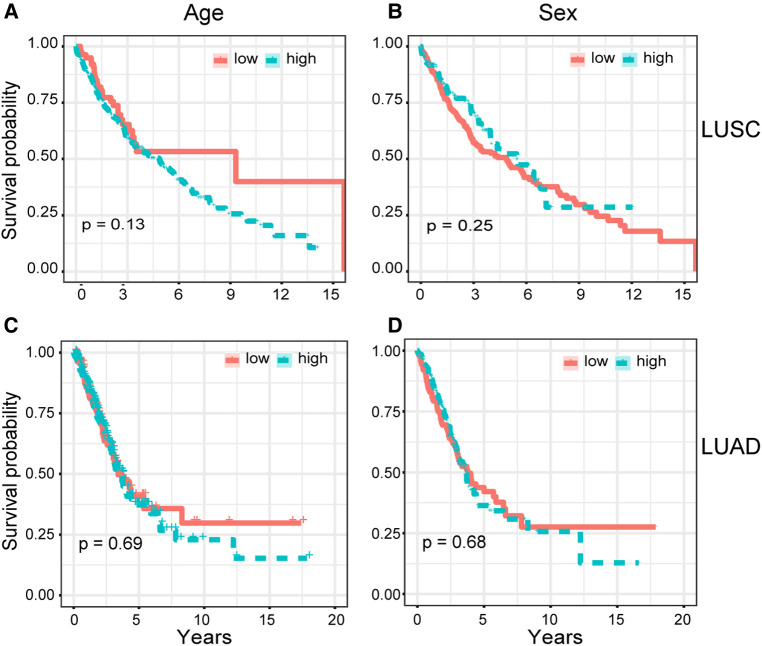
Cox univariate analysis. Relationships between clinical characteristics and patients' overall survival rate were shown. (**A,B**) show the relationship between patients' overall survival rate with age and sex for LUSC, respectively. (**C,D**) show the relationship between patients' overall survival rate with age and sex for LUAD, respectively.

**Figure 6:**
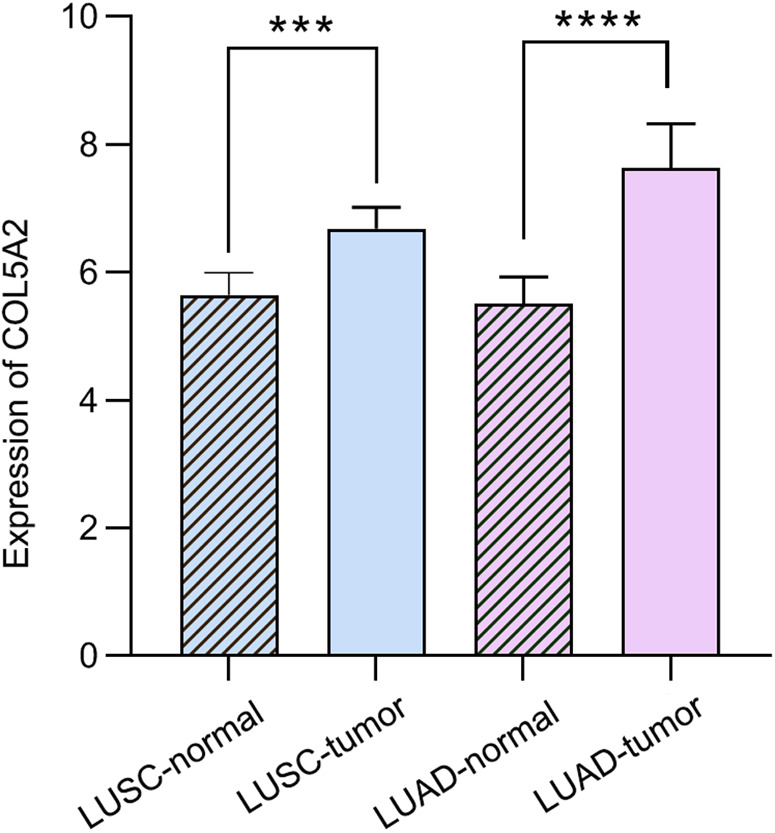
The relative expression of *COL5A2*. Stars represent statistical significance of *T*-test: ***<0.001; ****<0.0001.

To determine whether the *COL5A2* gene is a major prognostic factor, we correlated the clinical data of LUSC and LUAD with patient prognoses (Table 2). The survival analysis results showed that age and gender have no significant relationship with LUSC and LUAD patients' survival rates (Figure 5). Moreover, the *COL5A2* gene expression in LUSC and LUAD samples is significantly higher than that in normal samples (*P* < 0.001, Figure 6). Therefore, *COL5A2* may serve as a potential therapeutic target for NSCLC.

**Table 2:** LUSC and LUAD clinical characteristics.

Covariate	Category	Quantity	
		LUSC	LUAD
Age	<60	88	138
	≥60	388	357
Gender	Male	358	239
	Female	127	275

### *COL5A2* subnetwork

**Figure 7:**
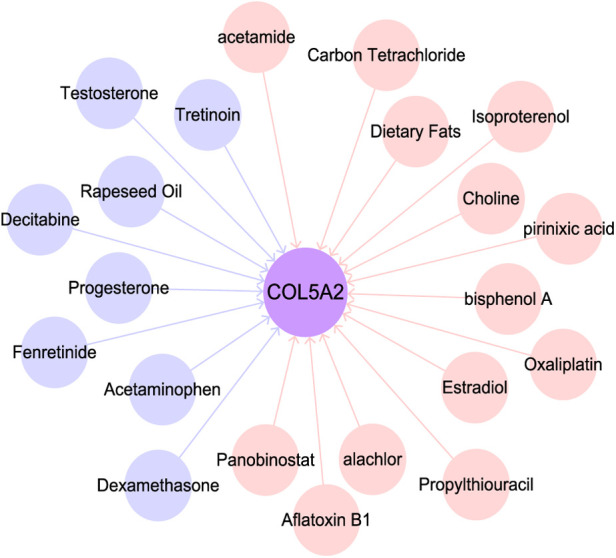
Substances related to *COL5A2*. Pink and blue circles represent substances that up- and downregulate *COL5A2*.

To investigate the role of *COL5A2*, we searched the CTD database. We found that *COL5A2* may regulate chemicals and human diseases and it is related to tumor treatment drugs. In addition, many tumor-related substances or drugs can affect the expression of *COL5A2* (Figure 7). For example, PIRINIXIC acid can increase *COL5A2* expression by binding to PAPAYA protein and increasing its activity. In addition, tumor preventive drugs such as decitabine and fenretinide can downregulate *COL5A2*. Dexamethasone can reduce *COL5A2* expression and suppresses the testosterone response.

## Discussion

We used a network approach to find a number of highly interconnected transcriptional events associated with non-small cell lung cancer (Figure 1). Our findings provided a preliminary view of the regulatory landscape of LUSC and LUAD. For LUSC, some genes, such as *AKR1C1*, *AKR1C2*, and *AKR1C3*, are enriched in the network module for regulating cell growth pathways. Overexpression of *AKR1C1* is associated with cancer progression (25). In addition, *AKR1C1* can directly interact with and promote phosphorylation of *STAT3*, enhancing the binding of *STAT3* to the promoter regions of target genes, and then transactivating these genes, promoting tumor metastasis (26). Therefore, further studies on the mechanisms of *AKR1C1*, *AKR1C2*, and *AKR1C3* in LUSC may provide new candidate targets for the treatment of patients.

Furthermore, genes *CCL19*, *CCR7*, *CCL21*, and *LY9* are enriched in LUAD network modules of T-lymphocyte-related pathways, such as T-cell activation, regulation of lymphocyte activation, and regulation of T-cell activation. *CCL19*, *CCL21*, and *CCR7* are involved in inducing the directed migration of T lymphocytes to lymph nodes in LUAD. *CCL19* and *CCL21* are chemokines and *CCR7* is their receptor in gastric cancer (27) and esophageal squamous cell carcinoma (28). The three genes play an important role in cell migration and lymph node metastasis (29). These chemokines may play a crucial role in directing immune cell migration, which is required to initiate an effective antitumor immune response (30).

Modules with high retention rates between LUSC and LUAD are enriched with similar functionality (Figures 1–3). For example, most LUSC modules show significant overlap with at least one LUAD module in terms of functionality (Table 1). LUSC and LUAD networks were found to share 46 common hub genes, of which *COL5A2*, *TTLL3*, *SPEF1*, *TMEM190*, *CCDC65*, *CCDC33*, and *GLT8D2* had the highest correlations with patient survival time. Further results showed that the *COL5A2* gene was highly expressed in both cancer subtypes with significantly poorer prognoses (Figure 6). *COL5A2*, encoding type V collagen a2, is upregulated in rapidly proliferating cells (31). In addition, *COL5A2* is involved in the occurrence and development of various malignancies, such as lung cancer (32), squamous cell carcinomas (33), bladder cancer (34), and colon cancer (35). We found that higher *COL5A2* expression was associated with lower survival in LUSC and LUAD patients (Figure 4). Our findings on *COL5A2* are largely consistent with previous studies that the *COL5A2* gene can be used to assess and predict prognosis in LUAD. Therefore, *COL5A2* may be a major factor in poor prognosis in LUSC and LUAD (Figure 7).

Considering that the pathogenesis of non-small cell lung cancer is still under investigation, we do not claim that our network approach can identify key genes in all classes of LUSC and LUAD, although it successfully found some instances with similar characteristics to those reported in the experiments. For further research, the following issues are worth: (1) investigating the utility and feasibility of *COL5A2* as a clinical marker; and (2) identifying the pathways in which *COL5A2* is involved and the key mechanisms that may guide personalized therapeutic strategies.

## Conclusion

LUSC and LUAD share a common network pattern, i.e., similar gene expression trends. *AKR1C1*, *AKR1C2*, and *AKR1C3* are enriched in the network module of LUSC for regulating cell growth pathways. *CCL19*, *CCR7*, *CCL21*, and *LY9* are keys in LUAD network modules of T-lymphocyte-related pathways. Furthermore, *COL5A2* may be a major factor in poor prognosis in LUSC and LUAD. The above findings may provide potential target genes for the early diagnosis of NSCLC and provide a new reference for the targeted therapy of LUSC and LUAD.

## Data Availability

The original contributions presented in the study are included in the article/**Supplementary Material**, further inquiries can be directed to the corresponding authors.
