# Supplementary material for: Shared network pattern of lung squamous carcinoma and adenocarcinoma illuminates therapeutic targets for non-small cell lung cancer
Source: Front Surg. 2022 Oct 3;9:958479. doi: 10.3389/fsurg.2022.958479 (PMC9576184; doi:10.3389/fsurg.2022.958479)
Supplement: Supplementary file 1 [file Table1.docx]

Table S1. LUSC and LUAD sample

| LUAD sample | LUSC sample |
| --- | --- |
| TCGA.05.4244.01 | TCGA-18-3407-01 |
| TCGA.05.4249.01 | TCGA-18-3414-01 |
| TCGA.05.4402.01 | TCGA-18-3415-01 |
| TCGA.05.4405.01 | TCGA-18-4086-01 |
| TCGA.05.4410.01 | TCGA-21-1071-01 |
| TCGA.05.4426.01 | TCGA-21-1076-01 |
| TCGA.05.4430.01 | TCGA-21-1082-01 |
| TCGA.38.4626.01 | TCGA-22-0940-01 |
| TCGA.38.6178.01 | TCGA-22-1000-01 |
| TCGA.38.7271.01 | TCGA-22-1002-01 |
| TCGA.38.A44F.01 | TCGA-22-1011-01 |
| TCGA.44.2657.01 | TCGA-22-1016-01 |
| TCGA.44.2659.01 | TCGA-22-4595-01 |
| TCGA.44.2666.01 | TCGA-22-4604-01 |
| TCGA.44.3918.01 | TCGA-22-4605-01 |
| TCGA.44.3919.01 | TCGA-22-4609-01 |
| TCGA.44.4112.01 | TCGA-22-5472-01 |
| TCGA.44.5645.01 | TCGA-22-5473-01 |
| TCGA.44.6148.01 | TCGA-22-5474-01 |
| TCGA.44.6775.01 | TCGA-33-4547-01 |
| TCGA.44.6777.01 | TCGA-34-2608-01 |
| TCGA.44.7659.01 | TCGA-34-5232-01 |
| TCGA.44.7672.01 | TCGA-34-5241-01 |
| TCGA.44.8120.01 | TCGA-34-5928-01 |
| TCGA.44.A47A.01 | TCGA-34-8454-01 |
| TCGA.44.A47G.01 | TCGA-34-8455-01 |
| TCGA.44.A4SU.01 | TCGA-39-5019-01 |
| TCGA.49.4490.01 | TCGA-39-5021-01 |
| TCGA.49.4501.01 | TCGA-39-5022-01 |
| TCGA.49.4505.01 | TCGA-39-5031-01 |
| TCGA.49.4512.01 | TCGA-39-5039-01 |
| TCGA.49.6744.01 | TCGA-43-3394-01 |
| TCGA.49.AAQV.01 | TCGA-43-6647-01 |
| TCGA.49.AAR4.01 | TCGA-43-6770-01 |
| TCGA.49.AARN.01 | TCGA-43-6773-01 |
| TCGA.49.AARO.01 | TCGA-43-8115-01 |
| TCGA.49.AARR.01 | TCGA-43-8118-01 |
| TCGA.50.5045.01 | TCGA-46-3765-01 |
| TCGA.50.5049.01 | TCGA-46-3766-01 |
| TCGA.50.5055.01 | TCGA-46-3767-01 |
| TCGA.50.5935.01 | TCGA-51-4079-01 |
| TCGA.50.5941.01 | TCGA-52-7622-01 |
| TCGA.50.5942.01 | TCGA-52-7811-01 |
| TCGA.50.5944.01 | TCGA-56-5897-01 |
| TCGA.50.6593.01 | TCGA-56-5898-01 |
| TCGA.50.8457.01 | TCGA-56-6545-01 |
| TCGA.50.8459.01 | TCGA-56-7731-01 |
| TCGA.50.8460.01 | TCGA-56-8308-01 |
| TCGA.53.7626.01 | TCGA-56-8503-01 |
| TCGA.55.1595.01 | TCGA-56-8625-01 |
| TCGA.55.6543.01 | TCGA-56-8626-01 |
| TCGA.55.6971.01 | TCGA-56-8628-01 |
| TCGA.55.6979.01 | TCGA-56-A4BW-01 |
| TCGA.55.6980.01 | TCGA-56-A4ZJ-01 |
| TCGA.55.6981.01 | TCGA-56-A5DS-01 |
| TCGA.55.6982.01 | TCGA-58-8386-01 |
| TCGA.55.6983.01 | TCGA-58-8393-01 |
| TCGA.55.6985.01 | TCGA-60-2698-01 |
| TCGA.55.6986.01 | TCGA-60-2704-01 |
| TCGA.55.6987.01 | TCGA-60-2707-01 |
| TCGA.55.7227.01 | TCGA-60-2708-01 |
| TCGA.55.7281.01 | TCGA-60-2709-01 |
| TCGA.55.7283.01 | TCGA-60-2710-01 |
| TCGA.55.7573.01 | TCGA-60-2713-01 |
| TCGA.55.7574.01 | TCGA-60-2721-01 |
| TCGA.55.7576.01 | TCGA-60-2723-01 |
| TCGA.55.7903.01 | TCGA-60-2725-01 |
| TCGA.55.7907.01 | TCGA-63-7020-01 |
| TCGA.55.7911.01 | TCGA-63-7021-01 |
| TCGA.55.8087.01 | TCGA-63-7022-01 |
| TCGA.55.8090.01 | TCGA-63-A5MG-01 |
| TCGA.55.8091.01 | TCGA-63-A5MP-01 |
| TCGA.55.8096.01 | TCGA-63-A5MS-01 |
| TCGA.55.8097.01 | TCGA-63-A5MV-01 |
| TCGA.55.8206.01 | TCGA-66-2727-01 |
| TCGA.55.8207.01 | TCGA-66-2734-01 |
| TCGA.55.8208.01 | TCGA-66-2737-01 |
| TCGA.55.8299.01 | TCGA-66-2742-01 |
| TCGA.55.8301.01 | TCGA-66-2753-01 |
| TCGA.55.8302.01 | TCGA-66-2758-01 |
| TCGA.55.8510.01 | TCGA-66-2765-01 |
| TCGA.55.8512.01 | TCGA-66-2767-01 |
| TCGA.55.8513.01 | TCGA-66-2770-01 |
| TCGA.55.8614.01 | TCGA-66-2773-01 |
| TCGA.55.8616.01 | TCGA-66-2777-01 |
| TCGA.55.8619.01 | TCGA-66-2780-01 |
| TCGA.55.8621.01 | TCGA-66-2781-01 |
| TCGA.55.A48X.01 | TCGA-66-2782-01 |
| TCGA.55.A48Z.01 | TCGA-66-2786-01 |
| TCGA.55.A4DG.01 | TCGA-66-2788-01 |
| TCGA.55.A57B.01 | TCGA-66-2789-01 |
| TCGA.62.8394.01 | TCGA-66-2791-01 |
| TCGA.62.A46R.01 | TCGA-66-2795-01 |
| TCGA.62.A46Y.01 | TCGA-66-2800-01 |
| TCGA.62.A472.01 | TCGA-68-7756-01 |
| TCGA.64.1680.01 | TCGA-68-A59I-01 |
| TCGA.64.1681.01 | TCGA-68-A59J-01 |
| TCGA.64.5815.01 | TCGA-77-6844-01 |
| TCGA.67.3772.01 | TCGA-77-7142-01 |
| TCGA.67.4679.01 | TCGA-77-7335-01 |
| TCGA.67.6215.01 | TCGA-77-7338-01 |
| TCGA.67.6216.01 | TCGA-77-8008-01 |
| TCGA.67.6217.01 | TCGA-77-8131-01 |
| TCGA.69.7765.01 | TCGA-77-8136-01 |
| TCGA.69.7974.01 | TCGA-77-8138-01 |
| TCGA.69.7980.01 | TCGA-77-8145-01 |
| TCGA.69.8254.01 | TCGA-85-6175-01 |
| TCGA.69.8453.01 | TCGA-85-6561-01 |
| TCGA.73.4658.01 | TCGA-85-7697-01 |
| TCGA.73.4662.01 | TCGA-85-7698-01 |
| TCGA.75.6203.01 | TCGA-85-7844-01 |
| TCGA.75.7025.01 | TCGA-85-8048-01 |
| TCGA.75.7030.01 | TCGA-85-8049-01 |
| TCGA.78.7143.01 | TCGA-85-8276-01 |
| TCGA.78.7147.01 | TCGA-85-8287-01 |
| TCGA.78.8648.01 | TCGA-85-8350-01 |
| TCGA.78.8655.01 | TCGA-85-8353-01 |
| TCGA.78.8660.01 | TCGA-85-8355-01 |
| TCGA.80.5607.01 | TCGA-85-8580-01 |
| TCGA.86.6562.01 | TCGA-85-8582-01 |
| TCGA.86.6851.01 | TCGA-85-A511-01 |
| TCGA.86.7714.01 | TCGA-90-6837-01 |
| TCGA.86.7954.01 | TCGA-90-7964-01 |
| TCGA.86.8055.01 | TCGA-92-7340-01 |
| TCGA.86.8056.01 | TCGA-94-A5I4-01 |
| TCGA.86.8074.01 | TCGA-96-7544-01 |
| TCGA.86.8075.01 | TCGA-96-7545-01 |
| TCGA.86.8076.01 | TCGA-96-8169-01 |
| TCGA.86.8278.01 | TCGA-96-8170-01 |
| TCGA.86.8280.01 | TCGA-98-8021-01 |
| TCGA.86.8668.01 | TCGA-98-8022-01 |
| TCGA.86.8671.01 | TCGA-98-A539-01 |
| TCGA.86.A456.01 | TCGA-98-A53B-01 |
| TCGA.86.A4P7.01 | TCGA-L3-A4E7-01 |
| TCGA.86.A4P8.01 | TCGA-NK-A5CX-01 |
| TCGA.91.6835.01 | TCGA-XC-AA0X-01 |
| TCGA.91.8497.01 |  |
| TCGA.93.7347.01 |  |
| TCGA.93.7348.01 |  |
| TCGA.93.A4JN.01 |  |
| TCGA.93.A4JO.01 |  |
| TCGA.93.A4JP.01 |  |
| TCGA.93.A4JQ.01 |  |
| TCGA.95.8039.01 |  |
| TCGA.95.A4VN.01 |  |
| TCGA.97.7546.01 |  |
| TCGA.97.7552.01 |  |
| TCGA.97.7553.01 |  |
| TCGA.97.7554.01 |  |
| TCGA.97.7938.01 |  |
| TCGA.97.7941.01 |  |
| TCGA.97.8172.01 |  |
| TCGA.97.8175.01 |  |
| TCGA.97.8177.01 |  |
| TCGA.97.8552.01 |  |
| TCGA.97.A4LX.01 |  |
| TCGA.97.A4M0.01 |  |
| TCGA.97.A4M1.01 |  |
| TCGA.97.A4M2.01 |  |
| TCGA.97.A4M5.01 |  |
| TCGA.97.A4M6.01 |  |
| TCGA.97.A4M7.01 |  |
| TCGA.99.7458.01 |  |
| TCGA.99.8025.01 |  |
| TCGA.99.8028.01 |  |
| TCGA.99.AA5R.01 |  |
| TCGA.J2.8192.01 |  |
| TCGA.J2.A4AE.01 |  |
| TCGA.J2.A4AG.01 |  |
| TCGA.L4.A4E6.01 |  |
| TCGA.L9.A444.01 |  |
| TCGA.L9.A743.01 |  |
| TCGA.MN.A4N5.01 |  |
| TCGA.MP.A4SV.01 |  |
| TCGA.MP.A4SW.01 |  |
| TCGA.MP.A4SY.01 |  |
| TCGA.MP.A4T4.01 |  |
| TCGA.MP.A4T9.01 |  |
| TCGA.MP.A4TH.01 |  |
| TCGA.MP.A4TJ.01 |  |
| TCGA.MP.A4TK.01 |  |
| TCGA.NJ.A4YG.01 |  |
| TCGA.NJ.A55A.01 |  |
| TCGA.S2.AA1A.01 |  |
