# Supplementary material for: Shared network pattern of lung squamous carcinoma and adenocarcinoma illuminates therapeutic targets for non-small cell lung cancer
Source: Front Surg. 2022 Oct 3;9:958479. doi: 10.3389/fsurg.2022.958479 (PMC9576184; doi:10.3389/fsurg.2022.958479)
Supplement: Supplementary file 2 [file Table2.docx]

Table S2. LUSC and LUAD network module overlapping genes number

| LUSC  LUAD | blue | brown | green | red | turquoise | yellow |
| --- | --- | --- | --- | --- | --- | --- |
| black | 5 | 12 | 0 | 11 | 36 | 9 |
| blue | 12 | 16 | 0 | 60 | 8 | 152 |
| brown | 254 | 11 | 0 | 5 | 9 | 1 |
| green | 5 | 4 | 137 | 0 | 40 | 2 |
| greenyellow | 0 | 6 | 0 | 0 | 37 | 0 |
| magenta | 12 | 49 | 0 | 13 | 5 | 0 |
| pink | 2 | 5 | 1 | 3 | 19 | 3 |
| purple | 1 | 1 | 2 | 0 | 12 | 1 |
| red | 9 | 13 | 3 | 5 | 20 | 4 |
| turquoise | 1 | 3 | 0 | 0 | 295 | 0 |
| yellow | 2 | 102 | 3 | 0 | 17 | 0 |

Note: The column name, the LUSC module color; the row name, the LUAD module color; LUSC, lung squamous cell carcinoma; LUAD, lung adenocarcinoma.
