# Supplementary material for: Shared network pattern of lung squamous carcinoma and adenocarcinoma illuminates therapeutic targets for non-small cell lung cancer
Source: Front Surg. 2022 Oct 3;9:958479. doi: 10.3389/fsurg.2022.958479 (PMC9576184; doi:10.3389/fsurg.2022.958479)
Supplement: Supplementary file 3 [file Table3.docx]

Table S3. LUSC and LUAD module GO enrichment results

| LUSC |  | LUAD |  |
| --- | --- | --- | --- |
| black | transmission of nerve impulse | blue | extracellular matrix organization |
|  | regulation of membrane potential |  | extracellular structure organization |
|  | multicellular organismal signaling |  | collagen fibril organization |
|  | membrane depolarization |  | ossification |
|  | neuronal action potential |  | cartilage development |
| blue | extracellular matrix organization | brown | T cell activation |
|  | extracellular structure organization |  | lymphocyte differentiation |
|  | collagen fibril organization |  | regulation of lymphocyte activation |
|  | ossification |  | regulation of T cell activation |
|  | cartilage development |  | T cell differentiation |
| brown | extracellular matrix organization | green |  |
|  | extracellular structure organization | red | anterior/posterior pattern specification |
|  | collagen fibril organization |  | regionalization |
|  | ossification |  | pattern specification process |
|  | cartilage development |  | embryonic skeletal system development |
| green | axoneme assembly |  | embryonic skeletal system morphogenesis |
| greenyellow | aminoglycoside antibiotic metabolic process | turquoise | cilium movement |
|  | quinone metabolic process |  | axoneme assembly |
|  | tertiary alcohol metabolic process |  | microtubule bundle formation |
|  | glycoside metabolic process |  | cilium organization |
|  | regulation of extent of cell growth |  | microtubule-based movement |
| magenta | multicellular organismal signaling | yellow | respiratory gaseous exchange |
|  | extracellular structure organization |  |  |
|  | muscle contraction |  |  |
| pink | negative regulation of axonogenesis |  |  |
|  | regulation of axonogenesis |  |  |
|  | positive regulation of axonogenesis |  |  |
|  | regulation of cellular component size |  |  |
|  | epidermis development |  |  |
| purple | epidermis development |  |  |
|  | cornification |  |  |
|  | epidermal cell differentiation |  |  |
|  | keratinization |  |  |
|  | skin development |  |  |
| red | anterior/posterior pattern specification |  |  |
|  | regionalization |  |  |
|  | pattern specification process |  |  |
|  | synaptic vesicle clustering |  |  |
|  | neuromuscular process |  |  |
| turquoise | cilium movement |  |  |
|  | axoneme assembly |  |  |
|  | microtubule bundle formation |  |  |
|  | axonemal dynein complex assembly |  |  |
|  | cilium organization |  |  |
| yellow | surfactant homeostasis |  |  |
|  | plasminogen activation |  |  |
|  | chemical homeostasis within a tissue |  |  |
|  | positive regulation of protein secretion |  |  |
|  | positive regulation of secretion |  |  |
