# Supplementary figures and images for: Shared network pattern of lung squamous carcinoma and adenocarcinoma illuminates therapeutic targets for non-small cell lung cancer
Source: Front Surg. 2022 Oct 3;9:958479. doi: 10.3389/fsurg.2022.958479 (PMC9576184; doi:10.3389/fsurg.2022.958479)

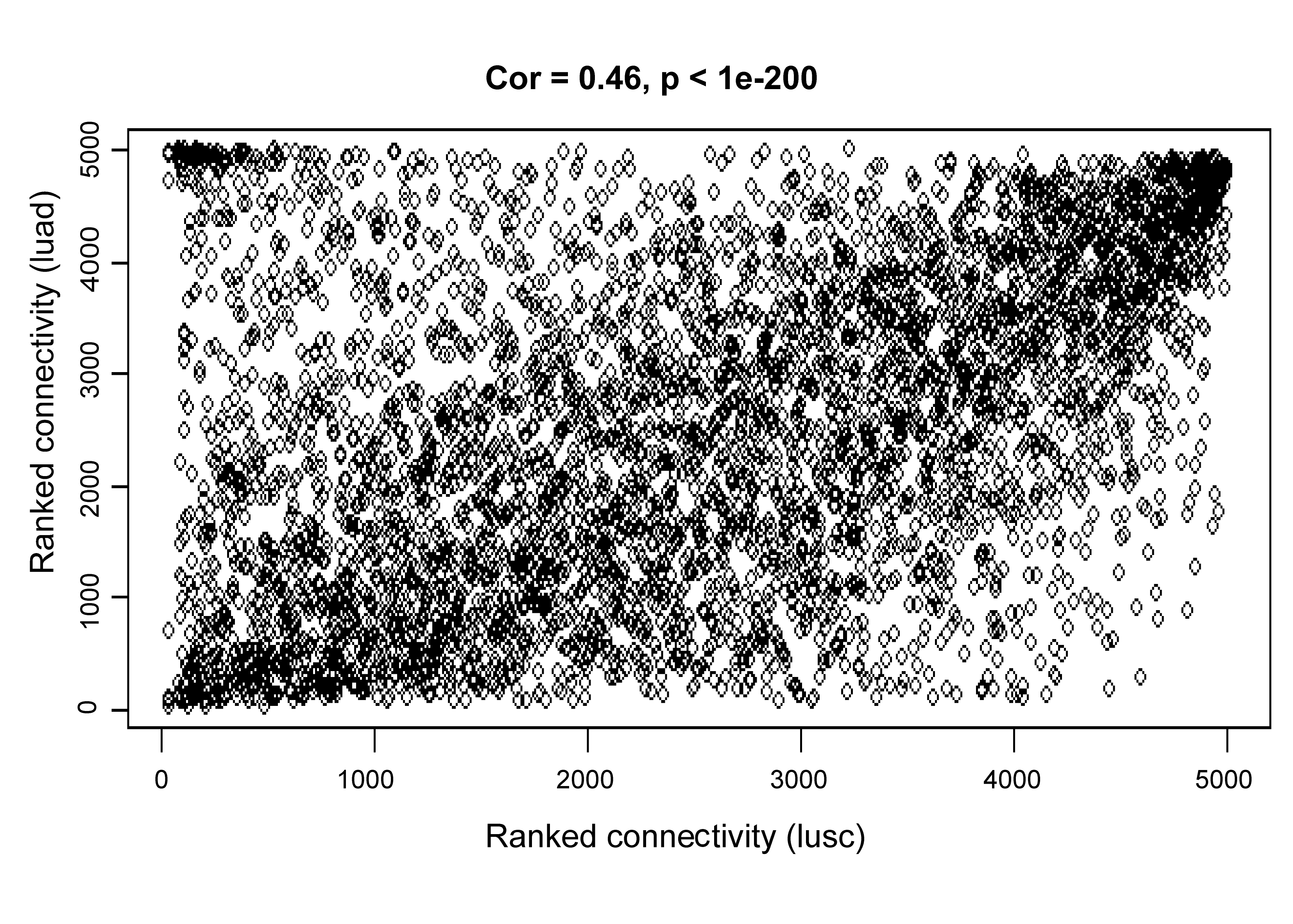

Supplement: Supplementary file 4 [file Image1.tif]

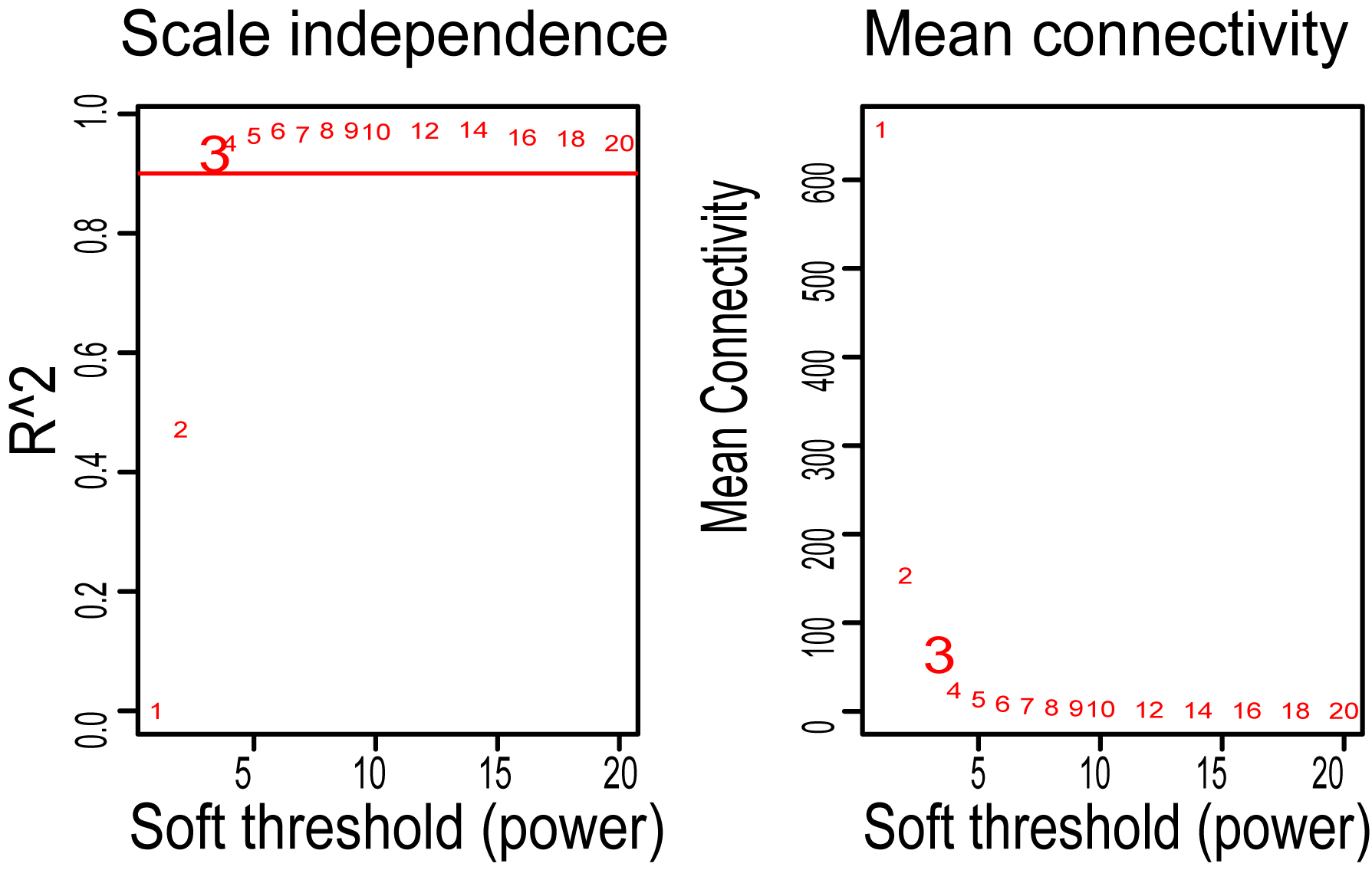

Supplement: Supplementary file 5 [file Image2.tif]
